# Uncertainty evaluation of data acquisition and analysis system relevant to infrared flowing medium laser

**DOI:** 10.1038/s41598-022-22667-2

**Published:** 2022-10-26

**Authors:** Rajeev Kumar Dohare, Mainuddin, Avinash C. Verma, Gaurav Singhal

**Affiliations:** 1 DIT&CS, DRDO, New Delhi, 110011 India; 2 Department of Electronics and Communication Engg., Jamia Millia Islamia, New Delhi, 110025 India; 3 CHESS, DRDO, Hyderabad, 500069 India

**Keywords:** Engineering, Optics and photonics

## Abstract

In flowing medium Chemical Oxygen Iodine Laser (COIL), Singlet oxygen is produced by the exothermic reaction of basic hydrogen peroxide solution and chlorine gas. It pumps the iodine and lasing process takes place by stimulated emission. Laser power is extracted using cavity. Development of customized data acquisition system is essential for measurements and analysis of both fundamental (temperature, pressure, level) as well as derived parameters (lasing medium concentration, flow rates of gases and laser power). The focus of the present paper is to dwell on uncertainty evaluation of a complex gas laser source in terms of ascertaining influences of primary/fundamental variables and corresponding derived parameters along with manner of uncertainty propagation. The study facilitates determining the variables with most significant impact on system performance, critical form point of view from optimal functioning of large-scale systems. This enables prediction of overall system uncertainty potentially extendable to other similar laser systems involving subsystems with mutual interdependencies together being distributed over a significantly large laboratory space. The relative combined uncertainty is computed to be 8.3%. The methodology shows significant potential for true decision-making and control of realistic gas laser source operation using developed 150 channel Data Acquisition and Analysis System (DAAS).

## Introduction

**Figure 1:**
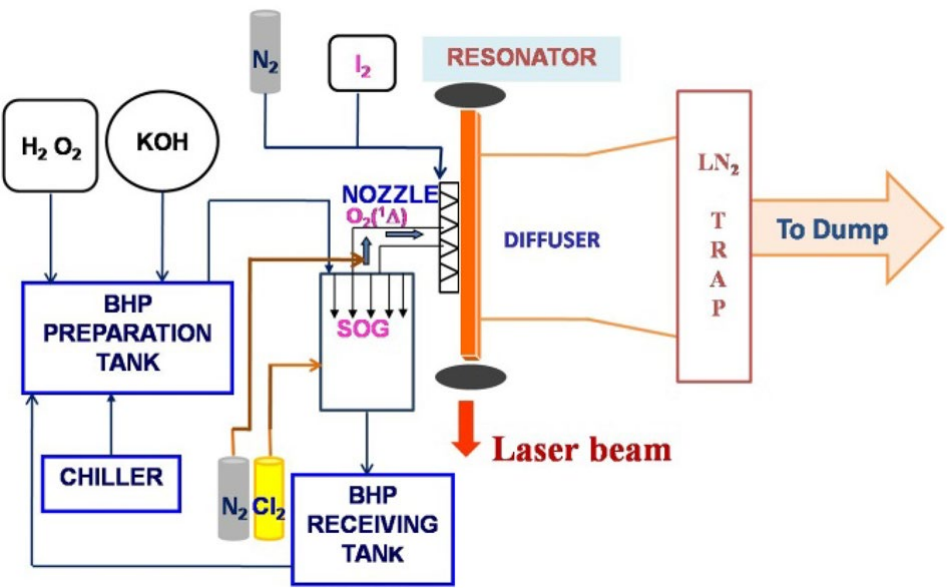
Basic COIL schematic.

The flowing medium gas lasers^1^ such as CO_2_ gas dynamic laser (λ = 10.6 µm)^2^, Hydrogen Fluoride–Deuterium Fluoride (HF-DF, λ = 2 0.7 − 3.4 µm^3^, and chemical oxygen iodine laser (COIL, λ = 1.315 µm^4,5^ emits in the infrared region. Among these lasers, COIL is scalable and offers wide range of applications with high efficiencies^6–9^ and good beam quality. Possibility of high-power, long-distance fiber delivery of the COIL laser makes it scalable to Mega Watt class level suitable for potential defense application. Figure 1 shows basic COIL schematic, which is typically a low pressure system operating at cavity pressures in range of 3–6 torr. In COIL, a reaction occurs between basic hydrogen peroxide (BHP) and chlorine gas (Cl_2_) to produce electronically excited singlet oxygen which is mixed with the lasing species stream i.e. (I_2_ + N_2_) inside the nozzle. The laser power is extracted at the laser cavity form the supersonic gas stream and the exhaust effluents are subsequently trapped in liquid nitrogen trap.

The optimum performance of COIL is governed by concentration of the lasing specie iodine, flow rates of nitrogen and chlorine, pressures in cavity, Pitot and plenum, iodine temperature, flow rates of circulating water in power measurement system (calorimeter) and BHP level. Monitoring and analysis of these critical gas dynamic parameters within desired accuracy and uncertainty limits is essential for precise control of COIL system. This is essential for characterization of several complex, obscure phenomenon occurring in the laser flow channel and to form requisite understanding for the same.

Uncertainty^10–12^ analysis is an effective process optimization tool in core research areas, which provides a good measure of realistic system performance and allows the user to identify key areas of improvement. In case of a COIL system it is obtained by evaluating parametric measurement uncertainty of both fundamental as well as derived parameters.

In order to evaluate errors and uncertainty in various measurements, International Organization for Standardization (ISO) has published the first edition of “Guide to the Expression of Uncertainty in Measurement (GUM)” [GUM^11^]. Also evaluation and expression of uncertainty is formally established for a wider range of measurements^12–14^. Uncertainty analysis of various systems has been reported by different researchers including diagnostics and wired data acquisition for COIL laser^15^ evaluation of expanded uncertainty using the LabVIEW in direct measurements^16^, error analysis of the data for evaluation of the performance of supersonic exhaust diffuser using scaled down models^17^, analysis of the projected errors of the chlorine utilization, water vapor pressure and singlet oxygen yield of the singlet oxygen chemical generator, equipped by the multichannel data acquisition system^18^, reliable uncertainty analysis and data evaluation for web based data acquisition system for poultry management^19^ and Type A and Type B uncertainty evaluation with improved methods^20^.

COIL (λ = 1315 nm) employs multidisciplinary technological areas for laser system realization. Development of customized data acquisition system is one of them. Typically, wired data acquisition systems^15,21^ have been mostly reported for operation of high power flowing medium laser systems on account of their reliability and proved track record. However, wired networks face issues pertaining to lack of accessibility and mobility. Further, they incur significant after-development trouble shooting costs due to various problems associated with cabled data acquisition system.

Hence, present development is focussed upon plausible alternative to the wired DAAS and explores wireless based technologies. This scheme of monitoring and control (data transmission) provides benefits in terms of reduction in size and involved installation/system costs, increased flexibility along with allowing simplified system deployments. Among the different wireless technologies available, Wi-Fi based systems are known to show better resistance to interference, reliable secure and better encryption and acceptable bit error rate of ~ 10^–9^. Also, it uses IEEE 802.11i security standard with 128 bit AES encryption. It also implements IEEE 802.1X, which is used for network authentication (Cranley, ITC). The systems also demonstrate high performance with regards to throughput, larger ranges, high data processing rates and high sampling rate (more bandwidth than ZigBee or Bluetooth). Further, power consumption of Wi-Fi based systems is low as compared to other such systems.

Hybrid data acquisition and analysis techniques utilize benefits of both wired and wireless technologies and have been successfully implemented in various practical real life scenarios like flight test networks^22^, smart grid applications^24^, monitoring of underground electricity substation^25^, monitoring of cultural heritage physical parameters^26^, greenhouse management^27^, turbine power generation system^28^. Hence, a hybrid DAAS appears to be an appropriate solution for use in a complex scenario such as flowing medium gas lasers and mitigating the shortfalls of a purely wired systems. Although researchers have developed data acquisition systems^21–27,29,30^ based on wired as well as hybrid techniques in different areas, but no one has reported detailed uncertainty analysis of data acquisition system for flowing laser to the best of our knowledge. The application of a hybrid DAAS for flowing medium gas laser and assessing its efficacy by employing methodical approach based on uncertainty evaluation, using GUM, forms the prime emphasis of the present work. The uncertainty analysis for flowing gas lasers has been briefly discussed primarily pertaining to gas flow parameters^15^, however, no specific studies with detailed uncertainty analysis aimed for entire high power gas laser system has been reported to the best of our knowledge. Although, accepted GUM practices of determining Type-A and Type-B uncertainties, relative uncertainties of derived parameters and overall combined uncertainty have been utilized, however the application of these concepts to a contemporary high power laser system makes it intricate primarily because similar analysis has not been largely reported in open literature.

In order to achieve realistic performance of COIL, a customized 150 channel hybrid concept-based Data Acquisition and Analysis System (DAAS) has been developed for remote operation of laser^31–33^. A structured approach utilizing detailed uncertainty evaluation has been used for ascertaining the effectiveness of the alternative DAAS system.

Overall operation of COIL laser generation process occurs within seconds (~ 10 s). Therefore, manual operation for such critical, complex and high end laser system is not viable. This is achieved by developed COIL DAAS, having 64 channels (analog inputs, AI) for parameter acquisition, 64 channels (digital outputs, DO) for valves (solenoid valve, electro-pneumatic valve, slit valve etc.) operation, 5 channels (analog outputs, AO) for gas feed and 17 channels (digital inputs, DI) for status monitoring of various valves/actuators. The parameter (variable) acquisition and analysis during the laser operation is an integral part of any data acquisition and analysis system pertaining to the safe sequential operation, required for successful laser operation. The focus primarily is to examine the efficacy of developed DAAS for multi variable, flowing medium COIL laser system.

Since gas laser systems, including COIL, are inherently complex involving several chemical processes, the system predictability and reliability becomes extremely subjective. In order to achieve repeatable optimal gas laser functioning for potential laser battlefield applications it is essential to characterize the uncertainty in laser functioning in terms of measurable macroscopic variables (primary/derived). It is in this context that a detailed uncertainty analysis and identification of most significant influences becomes critical. The uniqueness of the paper essentially lies in being able to identify these objective variables using the outlined uncertainty analysis. Further, it is quite evident that the control of these major influencing parameters is viable through custom DAAS instead of attempting to control the subtle chemical phenomenon occurring inside the laser flow field. Also, from application point of view this will enable in accurate prediction of scale of power utilized to beam potential targets producing soft kill/ hard kill and disabling target scenarios.

The analysis has been carried out by breaking down the system into direct parameter (pressure, temperature) and derived parameter (Flow rates, Gain etc.) measurement variables.

The temporal variation of most influencing variable (pressure) have been recorded in real time at various critical locations including Singlet Oxygen Generator (SOG), Cavity, Pitot, Plenum and gas/reagent flow systems. It enhances the decision making capability of the methodological approach and emerges out as an efficacious improvement tool for the measurement uncertainty evaluation.

The effects of the random sources are estimated by means of a multiple readings method and statistical approach (Type-A method for uncertainty estimation). On the other hand, uncertainty in observations other than repeated observations, *Type-B* uncertainty evaluation is carried out based on previous experiences and available information other than Type-A. Both the Type-A and Type-B uncertainties are combined to evaluate actual uncertainty and are referred as *combined uncertainty*. Relative uncertainty is defined either as measurement compared to the size of the measurement or as a percentage of the parameter under observation. The uncertainty, which results within the required samples percentage limit (confidence interval) is represented by the expanded uncertainty and is obtained by multiplying combined uncertainty by the coverage factor.

## Systems description

COIL is a short operating duration (~ 10 s) laser with subsystems distributed over a large area with significant physical distances thereby rendering manual operation infeasible. It also utilizes toxic and hazardous chlorine, iodine and BHP during its operation. Therefore, in order to operate COIL laser remotely with safety, development of a customized DAAS is of prime importance.

### DAAS architecture

**Figure 2:**
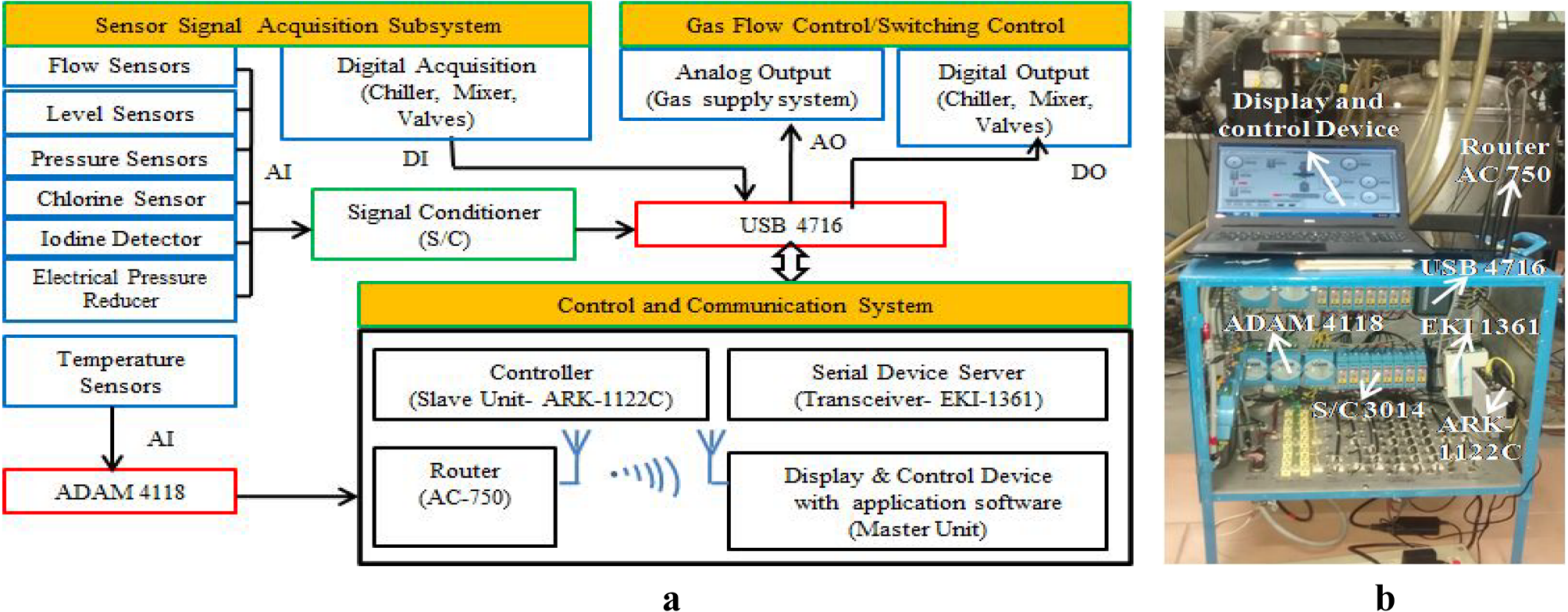
(**a**) Block diagram of DAAS architecture. (**b**) Developed DAAS.

A 150 channel DAAS comprising of 64 analog inputs (AI), 64 digital outputs (DO), 5 analog outputs (AO) and 17 digital inputs (DI) has been developed^31^. Main subsystems of developed DAAS are viz., (i) Sensor signal acquisition, (ii) Gas flow control/Switching control and (iii) Control and communication module. The block diagram of DAAS architecture is shown in Fig. 2a.

Sensor signal acquisition subsystem is responsible for acquiring different parameters such as temperature, pressure, flow rates and reagent level at different subsystems of COIL laser in analog input form (AI). It is also responsible for providing feedback with regards to status of various valves, chiller and other critical control modules of laser system in digital input form (DI).

Gas flow control/switching control (AO/DO) provides regulated gas supply to COIL system and is responsible for switching control of valves like solenoid valve/electro- pneumatic valve, mixer and chiller during laser finings for safe operation. Control and communication system comprises of Controller ARK 1122C, serial device server EKI-1361, router and display and control device with application software. Figure 2b shows actual developed DAAS hardware system for COIL.

DAAS has been configured using a 16 bit multifunction card USB 4716 (range 0–10 V). It has 16 single-ended or 8 differential channels AI, 2 channels of AO, 8 TTL channels of DI, 8 TTL channels of DO. Signal conditioner (S/C, Advantech 3014) provides optical isolation for protecting signals from harmful effects of ground loop, motor noise and other interferences. The features of the sensors employed in DAAS for COIL operation are listed in Table 1.

**Table 1:** Features of the sensors/detectors used in COIL operation using DAAS.

Sensor	Type	Model no	Manufacturer	Measurement range	Accuracy (Of Full Scale Range, FSR)
Pressure sensor	Stainless diffused silicon	YD 322A	Xían Yunyi Instruments Co. Ltd, China	(0–10) kPa (Cavity, Pitot, Plenum) (0–0.1) MPa (Chlorine gas)	± 0.1% (of FSR)
Iodine temperature sensor	K type thermocouple	Customized	Senstech Sensors, India	(273–473) K	± 0.5% (of Full Scale Range-FSR)
BHP level sensor	Hydraulic	3536	Metran, Russia	0–16 kPa	± 0.5% (of FSR)
Electrical pressure reducer (EPR)	Electro-Pneumatic	ITV Series	SMC, India	(0.1–0.9) MPa (primary and secondary N_2_ gas)	± 0.2% (of FSR)
Flow meter	Turbine	RN3	Flow technik, UK	(1.2–10) lpm	± 0.5% of FSR
Iodine concentration detector	Silicon	OSD100-6	Centronic, UK	(400–1100) nm (corresponding output 0–200 mV)	± 0.5% (over area)
Chlorine detector	Electochemical	9206	Husaini Engineers Pvt. Ltd., India	(0–10) ppm	± 1% of FSR

#### LabVIEW based software demonstration of DAAS

**Figure 3:**
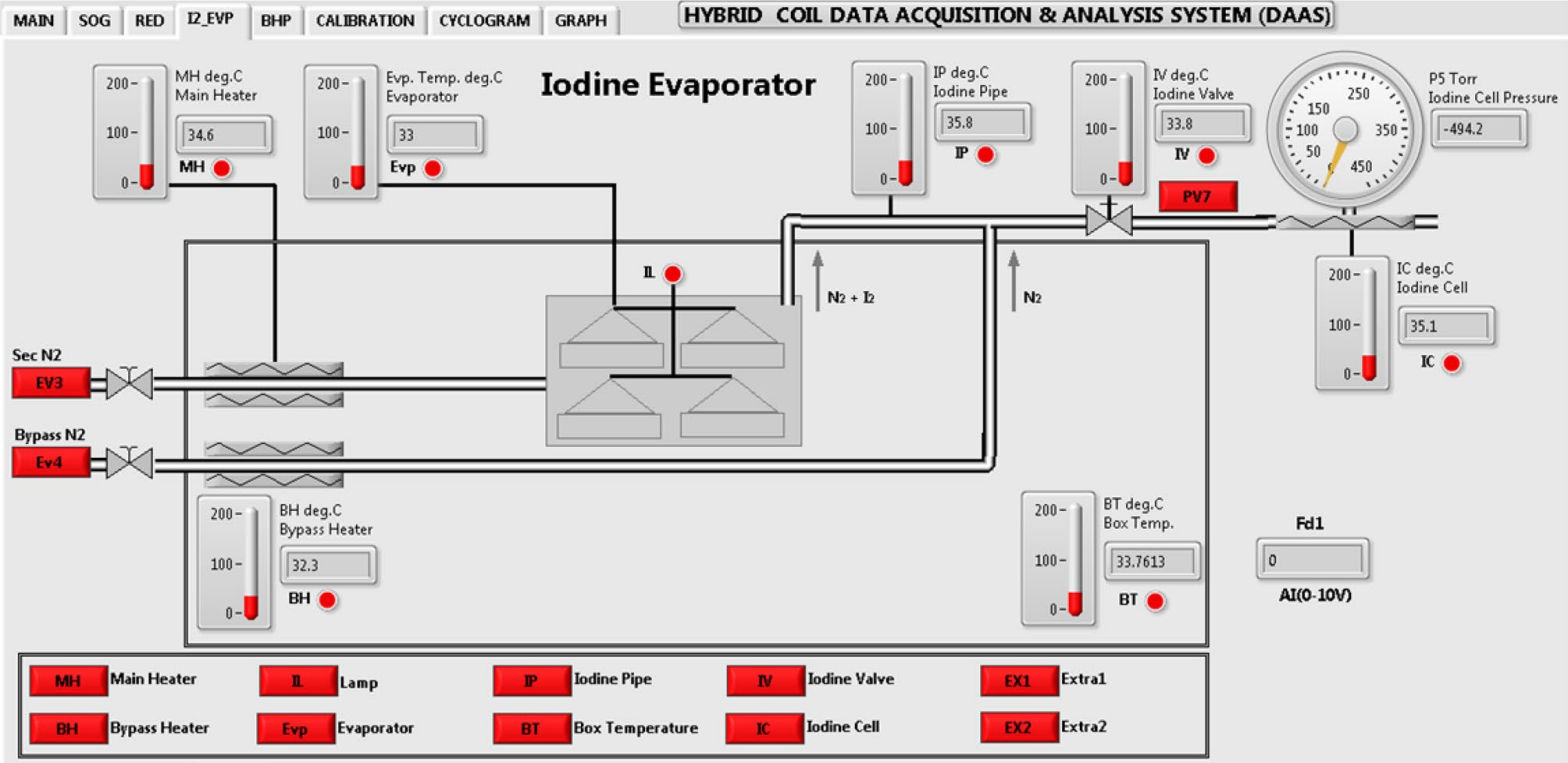
GUI for iodine evaporator system.

A LabVIEW platform based software program has been developed along with graphical user interfaces (GUI) for user friendly control and operation. Display and control device with application software is responsible for processing and execution of the program, display of various parameters indicating the real time status of different subsystems on the user interface. Eight GUIs have been developed for real time monitoring and control of different subsystem of COIL. A typical GUI for iodine evaporator module showing provisions for display of several pressures, temperatures, status of valve and their corresponding control is depicted in Fig. 3.

## Uncertainty analysis: methodology

The assessment of parameters with statistical theory is termed as Type A evaluation (obtained from frequency distributions), while assessment by any other means is termed as Type B evaluation (obtained from priori distribution)^10–14^. The various aspects of uncertainty analysis and computation of overall system uncertainty have been discussed in the present section. This mainly consists of estimating standard uncertainties attributable to direct sensor measurement, their relative uncertainties and similar uncertainty determinations for derived parameters. Subsequently, based on individual parametric uncertainties the variation in overall system performance is ascertained. It has been assumed that the variables in all the equations are independent and uncorrelated. Hence, a sum of squares method is used for all uncertainty calculations [GUM].

### Uncertainty analysis for sensors data (including fundamental parameters)

**Figure 4:**
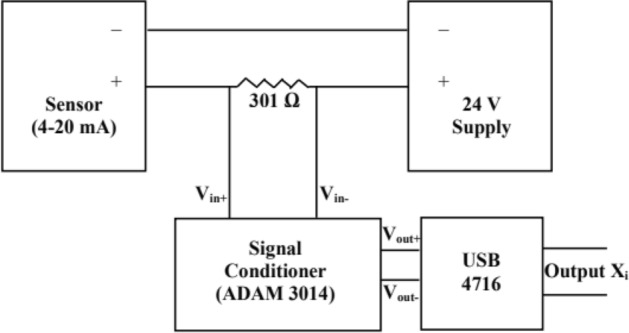
Sensor connection with signal conditioner.

Figure 4 shows sensor connection scheme with signal conditioner ADAM 3014 used for pressure, level and flow measurement channels. Output of the sensors (except temperature sensor) is 4–20 mA, which is converted into voltage signal using 301 Ω resistance. Hence, the input signal to ADAM 3014 is voltage signal. Detailed uncertainty evaluation has been carried out taking into account the potential uncertainties associated with each module in the line of sequence from sensor to display and control device for fundamental parameters by using statistical approach.

Propagation of uncertainty for different sensors (pressure, temperature, flow rate, level etc.) and DAAS signal cards (ADAM 3014, USB 4716 and ADAM 4118) is achieved using *Type-B uncertainty method* $u_{B} \left( {x_{i} } \right)$. This is evaluated by using accuracies (from manufacturers specifications) of all sensors and DAAS cards for parametric measurement at different subsystems (Iodine Evaporator, Nozzle, Cavity, Pitot, Plenum etc.) of flowing medium COIL laser. Overall error is evaluated by taking RSS of all errors during sensor signal processing.Evaluation of standard uncertainty for each parameter measurement is listed in Table 2.

**Table 2:** Evaluation of Standard uncertainty $u_{B} \left( {x_{i} } \right)$ by Type B method.

Sensor	Sensor error	Signal conditioner (ADAM 3014) Accuracy (of FSR)	DAAS signal cards USB 4716/ADAM 4118 Accuracy (of FSR)	Processing error	Reliability of measured parameters value (for 95% case)	Overall error (RSS of all errors, using Eq. 3)	Measurement value of FSR (F), (from Fig. 2)	Std. uncertainty $u_{B} \left( {x_{i} } \right)$ = F × overall error
Pressure	± 0.1%	± 0.1%	± 0.05%	± 0.1%	0.27%	± 0.32%	10 kPa, 0.1 MPa	32 Pa, 320 Pa
Temperature	± 0.5%	± 0.1%	± 0.1%	± 0.1%	0.27%	± 0.59%	200 K	1.2 K
Level	± 0.5%	± 0.1%	± 0.1%	± 0.1%	0.27%	± 0.59%	16 kPa	0.09 kPa
EPR	± 0.2%	± 0.1%	± 0.05%	± 0.1%	0.27%	± 0.37%	0.8 MPa	0. 003 MPa
Flow Sensor	± 0.5%	± 0.1%	± 0.05%	± 0.1%	0.27%	± 0.59%	8.8 lpm	0.05 lpm
I_2_ Detector	± 0.5%	± 0.1%	± 0.05%	± 0.1%	0.27%	± 0.59%	200 mV	1.2** × **10^–3^ V
Cl_2_ Sensor	± 1%	± 0.1%	± 0.05%	± 0.1%	0.27%	± 1%	10 ppm	0.1 ppm

In order to achieve successful lasing in COIL, typical operating pressure inside cavity must lie within 263.1–658 Pa (2–5, torr), which is working pressure (in cavity) in COIL laser and optimum for laser extraction achieved after supersonic expansion in the nozzle. Hence, focus is limited only in the range of (263.1–658) Pa. Iodine supply temperature (Lasing medium in COIL) is (353 ± 5) K for optimum performance of the COIL system.

*Type- A uncertainty evaluation,* $u_{A} \left( {x_{i} } \right)$is based upon a sample of data and sample size of 1 kSamples/s/channel (using Signal Conditioner ADAM 3014 and USB 4716) and 12 samples/s/channel (using ADAM 4118) recorded for pressure and temperature respectively. Detailed computations of standard uncertainty and relative uncertainty have been carried out for both pressure and temperature (within desired limits of operation) and are summarized in Table 3.

**Table 3:** Standard uncertainty, $u_{A} \left( {x_{i} } \right) = \frac{S}{\sqrt n }$ and Relative uncertainty, *u (r)* in cavity pressure and iodine temperature measurement.

Parameter		Sample size (n) at DAAS	Mean value of multiple readings during experiments $\left( X \right)$	Sample standard deviation $\left( S \right)$	Standard uncertainty (Type A) $u_{A} \left( {x_{i} } \right)$	Relative uncertainty $u\left( r \right)$
P *(Xi)* (Pa)	263.1	1000 Samples	271	7.9	0.25	0.09%
	658.0	1000 Samples	663	5.0	0.16	0.02%
T *(Xi)* (K)	353	12 Samples	353.1	0.1	0.03	0.008%

Mean value of multiple readings (X) is evaluated as: $$\left( {X = {{\sum\nolimits_{i = 1}^{n} {X_{i} } } \mathord{\left/ {\vphantom {{\sum\nolimits_{i = 1}^{n} {X_{i} } } n}} \right. \kern-\nulldelimiterspace} n}} \right)$$ and standard deviation (S) is evaluated as: $$\left( {S = \sqrt {{\raise0.7ex\hbox{${\sum\nolimits_{i = 1}^{n} {\left( {X_{i} - X} \right)^{2} } }$} \!\mathord{\left/ {\vphantom {{\sum\nolimits_{i = 1}^{n} {\left( {X_{i} - X} \right)^{2} } } {n - 1}}}\right.\kern-\nulldelimiterspace} \!\lower0.7ex\hbox{${n - 1}$}}} } \right)$$.

### Uncertainty analysis of computed parameters

Uncertainty analysis of derived parameters^35^ is evaluated using functional relationship between derived and fundamental parameters. Assume that a derived parameter *y* is denoted by “Eq. (1)”,

1 $$y = f \left( {x_{1,} x_{2,} x_{3, \ldots } x_{N} } \right)$$

Uncertainty component in *y* due to the uncertainty in component *x* is expressed as “Eq. (2)”,

2 $$u(y_{i} ) = \left( {{\raise0.7ex\hbox{${\partial y}$} \!\mathord{\left/ {\vphantom {{\partial y} {\partial x_{i} }}}\right.\kern-\nulldelimiterspace} \!\lower0.7ex\hbox{${\partial x_{i} }$}}} \right)u\left( {x_{i} } \right)$$

where, $$\left( {{\raise0.7ex\hbox{${\partial y}$} \!\mathord{\left/ {\vphantom {{\partial y} {\partial x_{i} }}}\right.\kern-\nulldelimiterspace} \!\lower0.7ex\hbox{${\partial x_{i} }$}}} \right)$$ is the sensitivity in measurement of parameter *x*_*i*_.

Combined uncertainty of y for two dependent correlated input quantities *x*_*i*_ and *x*_*j*_according to the GUM is presented as “Eq. (3A)”.

3A $$u^{2} (y) = \sum\nolimits_{i = 1}^{N} {\sum\nolimits_{j = 1}^{N} {\frac{\partial f}{{\partial x_{i} }}} } \frac{\partial f}{{\partial x_{j} }}u(x_{i} ,x_{j} ).$$

With the assumption of that all the fundamental parameters are independent and uniformly distributed, “Eq. (3A)” can be rewritten as the Root Sum Square (RSS) of individual uncertainty components- “Eq. (3B)”,

3B $$u(y) = \sqrt {\sum\nolimits_{i = 1}^{N} {u\left( {y_{i} } \right)}^{2} } = \sqrt {\sum\nolimits_{i = 1}^{N} {\left[ {({\raise0.7ex\hbox{${\partial y}$} \!\mathord{\left/ {\vphantom {{\partial y} {\partial x_{i} }}}\right.\kern-\nulldelimiterspace} \!\lower0.7ex\hbox{${\partial x_{i} }$}})u\left( {x_{i} } \right)} \right]^{2} } } ,$$

where, *u*(*x*_*i*_) is uncertainty evaluated by Type A (by statistical means) or standard Type B (by any other means) and Relative uncertainty is expressed as “Eq. (4)”,

4 $$u\left( r \right) = {\raise0.7ex\hbox{${u\left( y \right)}$} \!\mathord{\left/ {\vphantom {{u\left( y \right)} y}}\right.\kern-\nulldelimiterspace} \!\lower0.7ex\hbox{$y$}} = \sqrt {\sum\nolimits_{i = 1}^{N} {\left[ {{{u\left( x \right)_{i} } \mathord{\left/ {\vphantom {{u\left( x \right)_{i} } {x_{i} }}} \right. \kern-\nulldelimiterspace} {x_{i} }}} \right]^{2} } } .$$

The relative uncertainty of various derived parameters such as flow rates for various buffer gases, chlorine and iodine concentration have been considered for evaluation using *relative uncertainty u(r)* methodology.

Nitrogen is used both as *primary and secondary buffer gas* in COIL. Primary buffer gas is mixed with singlet oxygen flow for reducing singlet oxygen self-quenching. Secondary buffer gas is used for mixing nitrogen with iodine. Chlorine is used for generation of singlet oxygen. *Iodine* is lasing specie or active medium in COIL. The laser performance is governed by flow rates of these gases during operation. The uncertainty in orifice diameter is governed by the accuracy of the measuring device, which in the present case is a Vernier caliper with a resolution (∆*d*_*0*_) of 0.02 mm.

Hence, uncertainty in area *u*_*A*_ is 3.1 × 10^–10^ m^2^, [By employing, {(*π/4*) × (∆*d*_*0*_)^2^}].

The orifice flow rate is governed by following relation, “Eq. (5)”, for choked flow condition operation of the orifice applicable in cases of primary and secondary nitrogen flows as well as chlorine flow,

5 $$\mathop m\limits^{.} = \sqrt {{\raise0.7ex\hbox{$\gamma $} \!\mathord{\left/ {\vphantom {\gamma R}}\right.\kern-\nulldelimiterspace} \!\lower0.7ex\hbox{$R$}}} \left\{ {{\raise0.7ex\hbox{${P_{0} A}$} \!\mathord{\left/ {\vphantom {{P_{0} A} {\sqrt {T_{0} } }}}\right.\kern-\nulldelimiterspace} \!\lower0.7ex\hbox{${\sqrt {T_{0} } }$}}} \right\}C_{d} \left[ {{\raise0.7ex\hbox{$2$} \!\mathord{\left/ {\vphantom {2 {\gamma + 1}}}\right.\kern-\nulldelimiterspace} \!\lower0.7ex\hbox{${\gamma + 1}$}}} \right]^{{{\raise0.7ex\hbox{${\gamma + 1}$} \!\mathord{\left/ {\vphantom {{\gamma + 1} {2\left( {\gamma - 1} \right)}}}\right.\kern-\nulldelimiterspace} \!\lower0.7ex\hbox{${2\left( {\gamma - 1} \right)}$}}}} ,$$

wherein, is the mass flow rate of gases, *γ* is the specific heat ratio, *P*_*0*_ and *T*_*0*_ are stagnation pressure and stagnation temperature respectively, *C*_*d*_ is the discharge coefficient (0.93), *A* is the orifice area and *R* is the characteristic gas constant. Specific heat ratio for the gases is exactly known on the basis of the gas employed, for N_2_, it is 1.4 and for Cl_2_, it is 1.33 and so on so forth. They are a weak function of pressure and vary largely only at high temperatures they may vary as encountered in cases of combustion, thereby they can be easily treated as constant. The *C*_*d*_ indicates the average discharge coefficient, which has been measured by employing a dedicated orifice characterization setup which plots the flow vs. upstream pressure taking several reading for each pressure value and the straight line plot gives the average expected flow rate for each pressure value for which theoretical flow rate is known. The ratio of the two gives us the average coefficient of discharge. Hence, both the specific heat ratio and discharge coefficient are assumed to be constant. Relative uncertainty in flow rate of Primary N_2_ and Cl_2_ using (4) is obtained as “Eq. (6)”, by taking partial differentiation of “Eq. (5)”, and listed in Table 4,

6 $${{u\left( {\mathop m\limits^{.} } \right)} \mathord{\left/ {\vphantom {{u\left( {\mathop m\limits^{.} } \right)} {\mathop m\limits^{.} }}} \right. \kern-\nulldelimiterspace} {\mathop m\limits^{.} }} = \sqrt {\left( {{\raise0.7ex\hbox{${u\left( {P_{0} } \right)}$} \!\mathord{\left/ {\vphantom {{u\left( {P_{0} } \right)} {P_{0} }}}\right.\kern-\nulldelimiterspace} \!\lower0.7ex\hbox{${P_{0} }$}}} \right)^{2} + \left( {{\raise0.7ex\hbox{${u\left( A \right)}$} \!\mathord{\left/ {\vphantom {{u\left( A \right)} A}}\right.\kern-\nulldelimiterspace} \!\lower0.7ex\hbox{$A$}}} \right)^{2} + \frac{1}{4}\left( {{\raise0.7ex\hbox{${u\left( {T_{0} } \right)}$} \!\mathord{\left/ {\vphantom {{u\left( {T_{0} } \right)} {T_{0} }}}\right.\kern-\nulldelimiterspace} \!\lower0.7ex\hbox{${T_{0} }$}}} \right)^{2} } .$$

**Table 4:** Relative uncertainty in Primary N_2_, Secondary N_2_ and chlorine measurement.

Parameter	Primary nitrogen		Secondary nitrogen		Chlorine	
	Measured value (x_i_)	Uncertainty in measured value $u\left( {x_{i} } \right)$ (from Fig. 2)	Measured value (x_i_)	Uncertainty in measured value $u\left( {x_{i} } \right)$ (from Fig. 2)	Measured Value (x_i_)	Uncertainty in measured value $u\left( {x_{i} } \right)$ (from Fig. 2)
Pressure, P_o_ (Pa)	0.45** × **10^6^	3** × **10^3^ (Using EPR)	1.3** × **10^5^	3** × **10^3^ (Using EPR)	8.9** × **10^4^	320
Temperature, T_o_ (K)	300	1.2	353	1.2	300	1.2
Orifice area, A (m^2^)	1.6** × **10^–5^	3.1** × **10^–10^	5** × **10^–5^	3.1** × **10^–10^	2.8** × **10^–5^	3.1** × **10^–10^
Relative uncertainty	0.7%		2.3%		0.4%	

Also, Molar flow rate variations may also be computed from “Eq. (7)”, by simplifying the “Eq. (5)”, using corresponding values of the constants as per the gases employed.

7 $$\begin{aligned} M_{c} & = \left\{ {{\raise0.7ex\hbox{${0.404P_{0} AC_{d} }$} \!\mathord{\left/ {\vphantom {{0.404P_{0} AC_{d} } {\sqrt {T_{0} } \left( {\frac{{0.028\,{\text{kg}}}}{{{\text{mol}}}}} \right)}}}\right.\kern-\nulldelimiterspace} \!\lower0.7ex\hbox{${\sqrt {T_{0} } \left( {\frac{{0.028\,{\text{kg}}}}{{{\text{mol}}}}} \right)}$}}} \right\}{\raise0.7ex\hbox{${{\text{kg}}}$} \!\mathord{\left/ {\vphantom {{{\text{kg}}} {\text{s}}}}\right.\kern-\nulldelimiterspace} \!\lower0.7ex\hbox{${\text{s}}$}} \\ & = \left\{ {{\raise0.7ex\hbox{${0.404P_{0} AC_{d} }$} \!\mathord{\left/ {\vphantom {{0.404P_{0} AC_{d} } {\sqrt {T_{0} } {{ \times }}0.028}}}\right.\kern-\nulldelimiterspace} \!\lower0.7ex\hbox{${\sqrt {T_{0} } {{ \times }}0.028}$}}} \right\}{\raise0.7ex\hbox{${{\text{mol}}}$} \!\mathord{\left/ {\vphantom {{{\text{mol}}} {\text{s}}}}\right.\kern-\nulldelimiterspace} \!\lower0.7ex\hbox{${\text{s}}$}} \\ \end{aligned}$$

The combined uncertainty, $u\left( {M_{c} } \right)$, using “Eq. (3B)”:

8 $$u\left( {M_{c} } \right) = \sqrt {\frac{{\left\{ {\left( {{\raise0.7ex\hbox{${\partial M_{c} }$} \!\mathord{\left/ {\vphantom {{\partial M_{c} } {\partial P_{0} }}}\right.\kern-\nulldelimiterspace} \!\lower0.7ex\hbox{${\partial P_{0} }$}}} \right)u_{{P_{0} }} } \right\}^{2} + \left\{ {\left( {{\raise0.7ex\hbox{${\partial M_{c} }$} \!\mathord{\left/ {\vphantom {{\partial M_{c} } {\partial T_{0} }}}\right.\kern-\nulldelimiterspace} \!\lower0.7ex\hbox{${\partial T_{0} }$}}} \right)u_{{T_{0} }} } \right\}^{2} }}{{ + \left\{ {\left( {{\raise0.7ex\hbox{${\partial M_{c} }$} \!\mathord{\left/ {\vphantom {{\partial M_{c} } {\partial A}}}\right.\kern-\nulldelimiterspace} \!\lower0.7ex\hbox{${\partial A}$}}} \right)u_{A} } \right\}^{2} }}}$$

Using partial derivatives in “Eq. (8)”, sensitivities are estimated as “Eq. (9)”

9 $${\raise0.7ex\hbox{${\partial M_{c} }$} \!\mathord{\left/ {\vphantom {{\partial M_{c} } {\partial P_{0} }}}\right.\kern-\nulldelimiterspace} \!\lower0.7ex\hbox{${\partial P_{0} }$}} = {\raise0.7ex\hbox{${M_{c} }$} \!\mathord{\left/ {\vphantom {{M_{c} } {P_{0} }}}\right.\kern-\nulldelimiterspace} \!\lower0.7ex\hbox{${P_{0} }$}},{\raise0.7ex\hbox{${\partial M_{c} }$} \!\mathord{\left/ {\vphantom {{\partial M_{c} } {\partial T_{0} }}}\right.\kern-\nulldelimiterspace} \!\lower0.7ex\hbox{${\partial T_{0} }$}} = - {\raise0.7ex\hbox{${M_{c} }$} \!\mathord{\left/ {\vphantom {{M_{c} } {2T_{0} }}}\right.\kern-\nulldelimiterspace} \!\lower0.7ex\hbox{${2T_{0} }$}},{\raise0.7ex\hbox{${\partial M_{c} }$} \!\mathord{\left/ {\vphantom {{\partial M_{c} } {\partial A}}}\right.\kern-\nulldelimiterspace} \!\lower0.7ex\hbox{${\partial A}$}} = {\raise0.7ex\hbox{${M_{c} }$} \!\mathord{\left/ {\vphantom {{M_{c} } A}}\right.\kern-\nulldelimiterspace} \!\lower0.7ex\hbox{$A$}}.$$

Sensitivities in “Eq. (9)” are evaluated by putting values of M_c_ {from “Eq. (7)”} and values of *P*_*0*_, *T*_*0*_ and *A* (from Table 4). Combined uncertainty for secondary nitrogen, $$u_{{M_{c} }}$$ is evaluated by using values of sensitivities and uncertainties of *P*_*0*_{*u*(*P*_*0*_)}, *T*_*0*_{*u*(*T*_*0*_)}, *A*{*u*(*A*)} (all from Table 4) and found to be, 1.1 × 10^–2^ mol/s. EPR provides controlled flow of secondary nitrogen by special software program developed using LabVIEW in developed DAAS. Relative uncertainty in secondary nitrogen flow rate measurement is estimated as: $${\raise0.7ex\hbox{${u\left( {M_{c} } \right)}$} \!\mathord{\left/ {\vphantom {{u\left( {M_{c} } \right)} {M_{c} }}}\right.\kern-\nulldelimiterspace} \!\lower0.7ex\hbox{${M_{c} }$}}$$ and listed in Table 4.

One of the most critical parameter influencing system performance is the *uncertainty in measurement of the lasing species (iodine)*. Iodine is available in solid crystal form at room temperature (25 °C). Hence it is required to be heated to ~ 80 °C (353 K) in order to obtain uniform iodine vapors in iodine evaporator system for achieving proper mixing of singlet oxygen and iodine vapors occurs in the supersonic nozzle. The stabilization of vapors at indicated temperature is obtained by using pulse width modulation (PWM) based temperature stabilization (Proportional derivative controller, PID, Model no. MC-2438-201-000, Make: Max. Thermo, Taiwan). The precipitation of iodine during transport before reaching COIL system is prevented by employing heated secondary nitrogen passed through the iodine evaporation system to serve as the carrier. An optical absorption based process is used for iodine concentration measurement.

**Figure 5:**
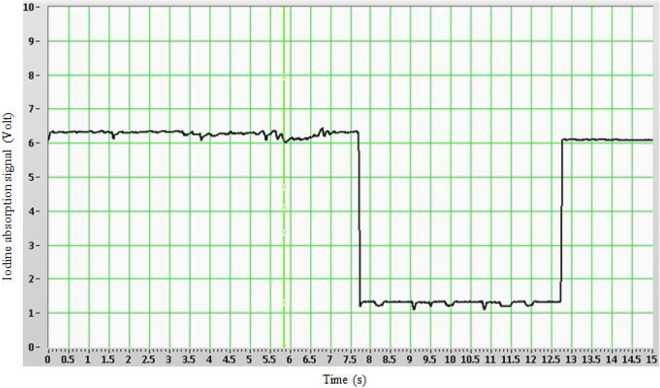
Temporal variation of Iodine absorption signal.

Iodine molar flow rate is expressed using the Beer Lambert law and the perfect gas law equation as “Eq. (10)”,

10 $$\dot{m}_{{I_{2} }} = \left\{ {\frac{{M_{c} kT_{0} }}{{\sigma _{v} L}}} \right\}\frac{{\ln \left( {\frac{{I_{0} }}{{I_{v} }}} \right)}}{{P_{{tot}} - \left( {\frac{{kT_{0} }}{{\sigma _{v} L}}} \right)\ln \left( {\frac{{I_{0} }}{{I_{v} }}} \right)}},$$

where *I*_*0*_ and *I*_*v*_ are light intensity in form of iodine detector signal without- upper lines and with iodine- bottom line (Fig. 5), σ_*v*_ is the absorption cross section (2.1 × 10^–22^ m^2^), *L* is the length of the iodine cell unit, *k* is the Boltzmann constant, *T* is the gas temperature, *P*_*tot*_ is the measured total pressure, *M*_*c*_ is the secondary N_2_ molar flow rate.

The sensitivity calculations using partial differentiation as given in “Eq. (4)” are:

11 $$\frac{{\partial \mathop {m_{{I_{2} }} }\limits^{.} }}{{\partial M_{c} }} = \left\{ {\frac{{kT_{0} }}{{\sigma_{v} L}}} \right\}\frac{{\ln \left( {\frac{{I_{0} }}{{I_{v} }}} \right)}}{{P_{tot} - \left( {\frac{{kT_{0} }}{{\sigma_{v} L}}} \right)\ln \left( {\frac{{I_{0} }}{{I_{v} }}} \right)}} = \frac{{\mathop {m_{{I_{2} }} }\limits^{.} }}{{M_{c} }},$$

12 $$\begin{aligned} \frac{{\partial \mathop {m_{{I_{2} }} }\limits^{.} }}{{\partial T_{0} }} & = \left[ {\left\{ {\frac{{M_{c} k}}{{\sigma _{v} L}}} \right\}\frac{{\ln \left( {\frac{{I_{0} }}{{I_{v} }}} \right)}}{{P_{{tot}} - \left( {\frac{{kT_{0} }}{{\sigma _{v} L}}} \right)\ln \left( {\frac{{I_{0} }}{{I_{v} }}} \right)}} + \left\{ {\frac{{M_{c} kT_{0} }}{{\sigma _{v} L}}} \right\}\ln \left( {\frac{{I_{0} }}{{I_{v} }}} \right) \times \left\{ {\frac{{ - 1}}{{\left\{ {P_{{tot}} - \left( {\frac{{kT_{0} }}{{\sigma _{v} L}}} \right)\ln \left( {\frac{{I_{0} }}{{I_{v} }}} \right)} \right\}^{2} }}} \right\} \times \left( {\frac{{ - k}}{{\sigma _{v} L}}} \right)\ln \left( {\frac{{I_{0} }}{{I_{v} }}} \right)} \right] \\ & = \frac{{\mathop {m_{{I_{2} }} }\limits^{.} }}{T}\left( {1 + \frac{{\mathop {m_{{I_{2} }} }\limits^{.} }}{{M_{c} }}} \right), \\ \end{aligned}$$

13 $$\frac{{\partial \mathop {m_{{I_{2} }} }\limits^{.} }}{{\partial L}} = \left[ \begin{gathered} \left\{ {\frac{{ - M_{c} kT_{0} }}{{\sigma _{v} L^{2} }}} \right\}\frac{{\ln \left( {\frac{{I_{0} }}{{I_{v} }}} \right)}}{{P_{{tot}} - \left( {\frac{{kT_{0} }}{{\sigma _{v} L}}} \right)\ln \left( {\frac{{I_{0} }}{{I_{v} }}} \right)}} + \left\{ {\frac{{M_{c} kT_{0} }}{{\sigma _{v} L}}} \right\}\ln \left( {\frac{{I_{0} }}{{I_{v} }}} \right)* \hfill \\ \left\{ {\frac{{ - 1}}{{\left\{ {P_{{tot}} - \left( {\frac{{kT_{0} }}{{\sigma _{v} L}}} \right)\ln \left( {\frac{{I_{0} }}{{I_{v} }}} \right)} \right\}^{2} }}} \right\} \times \left( {\frac{{ - kT_{0} }}{{\sigma _{v} }}} \right)\ln \left( {\frac{{I_{0} }}{{I_{v} }}} \right)\left( {\frac{{ - 1}}{{L^{2} }}} \right) \hfill \\ \end{gathered} \right] = - \frac{{\mathop {m_{{I_{2} }} }\limits^{.} }}{L}\left( {1 + \frac{{\mathop {m_{{I_{2} }} }\limits^{.} }}{{M_{c} }}} \right),$$

14 $$\frac{{\partial \mathop {m_{{I_{2} }} }\limits^{.} }}{{\partial P_{{tot}} }} = \left\{ {\frac{{M_{c} kT_{0} }}{{\sigma _{v} L}}} \right\}\ln \left( {\frac{{I_{0} }}{{I_{v} }}} \right) \times \left\{ {\frac{{ - 1}}{{\left\{ {P_{{tot}} - \left( {\frac{{kT_{0} }}{{\sigma _{v} L}}} \right)\ln \left( {\frac{{I_{0} }}{{I_{v} }}} \right)} \right\}^{2} }}} \right\} = - \frac{{\mathop {m_{{I_{2} }} }\limits^{.} }}{{\left\{ {P_{{tot}} - \left( {\frac{{kT_{0} }}{{\sigma _{v} L}}} \right)\ln \left( {\frac{{I_{0} }}{{I_{v} }}} \right)} \right\}}},$$

15 $$\frac{{\partial \mathop {m_{{I_{2} }} }\limits^{.} }}{{\partial I_{0} }} = \left\{ {\frac{{M_{c} kT_{0} }}{{\sigma _{v} L}}} \right\} \times \left[ {\frac{{\left\{ {P_{{tot}} - \left( {\frac{{kT_{0} }}{{\sigma _{v} L}}} \right)\ln \left( {\frac{{I_{0} }}{{I_{v} }}} \right)} \right\}\frac{1}{{\left( {{\raise0.7ex\hbox{${I_{0} }$} \!\mathord{\left/ {\vphantom {{I_{0} } {I_{v} }}}\right.\kern-\nulldelimiterspace} \!\lower0.7ex\hbox{${I_{v} }$}}} \right)}}\left( {\frac{1}{{I_{v} }}} \right) - \ln \left( {\frac{{I_{0} }}{{I_{v} }}} \right)\left( { - \frac{{kT_{0} }}{{\sigma _{v} L}}\frac{1}{{I_{0} }}} \right)}}{{\left\{ {P_{{tot}} - \left( {\frac{{kT_{0} }}{{\sigma _{v} L}}} \right)\ln \left( {\frac{{I_{0} }}{{I_{v} }}} \right)} \right\}^{2} }}} \right] = \frac{{\mathop {m_{{I_{2} }} }\limits^{.} }}{{I_{0} \ln \left( {\frac{{I_{0} }}{{I_{v} }}} \right)\,}}\left( {1 + \frac{{\mathop {m_{{I_{2} }} }\limits^{.} }}{{M_{c} }}} \right),$$

16 $$\begin{aligned} \frac{{\partial \mathop {m_{{I_{2} }} }\limits^{.} }}{{\partial I_{v} }} & = \left\{ {\frac{{M_{c} kT_{0} }}{{\sigma _{v} L}}} \right\} \times \left[ {\frac{{\left\{ {P_{{tot}} - \left( {\frac{{kT_{0} }}{{\sigma _{v} L}}} \right)\ln \left( {\frac{{I_{0} }}{{I_{v} }}} \right)} \right\} \times \left\{ {\frac{1}{{\left( {{\raise0.7ex\hbox{${I_{0} }$} \!\mathord{\left/ {\vphantom {{I_{0} } {I_{v} }}}\right.\kern-\nulldelimiterspace} \!\lower0.7ex\hbox{${I_{v} }$}}} \right)}}\left( { - \frac{{I_{0} }}{{I_{{v^{2} }} }}} \right)} \right\} - \left\{ {\ln \left( {\frac{{I_{0} }}{{I_{v} }}} \right)\left( { - \frac{{kT_{0} }}{{\sigma _{v} L}}} \right)\frac{1}{{\left( {{\raise0.7ex\hbox{${I_{0} }$} \!\mathord{\left/ {\vphantom {{I_{0} } {I_{v} }}}\right.\kern-\nulldelimiterspace} \!\lower0.7ex\hbox{${I_{v} }$}}} \right)}}\left( { - \frac{{I_{0} }}{{I_{{v^{2} }} }}} \right)} \right\}}}{{\left\{ {P_{{tot}} - \left( {\frac{{kT_{0} }}{{\sigma _{v} L}}} \right)\ln \left( {\frac{{I_{0} }}{{I_{v} }}} \right)} \right\}^{2} }}} \right] \\ & = - \frac{{\mathop {m_{{I_{2} }} }\limits^{.} }}{{I_{v} \ln \left( {\frac{{I_{0} }}{{I_{v} }}} \right)\,}}\left( {1 + \frac{{\mathop {m_{{I_{2} }} }\limits^{.} }}{{M_{c} }}} \right). \\ \end{aligned}$$

The experimental data for COIL system indicates that optimal iodine molar flow rate is 10 mmol s^−1^. By assuming negligible correlation among the variables in “Eq. (10)”,the combined uncertainty in iodine measurement using “Eq. (3B)” is calculated as “Eq. (17)”,

17 $$u\left( {\mathop {m_{{I_{2} }} }\limits^{.} } \right) = \sqrt {\left( {\frac{{\partial \mathop {m_{{I_{2} }} }\limits^{.} }}{{\partial M_{c} }}u\left( {M_{c} } \right)} \right)^{2} + \left( {\frac{{\partial \mathop {m_{{I_{2} }} }\limits^{.} }}{{\partial T_{0} }}u\left( {T_{0} } \right)} \right)^{2} + \left( {\frac{{\partial \mathop {m_{{I_{2} }} }\limits^{.} }}{\partial L}u\left( L \right)} \right)^{2} + \left( {\frac{{\partial \mathop {m_{{I_{2} }} }\limits^{.} }}{{\partial P_{tot} }}u\left( {P_{tot} } \right)} \right)^{2} + \left( {\frac{{\partial \mathop {m_{{I_{2} }} }\limits^{.} }}{{\partial I_{0} }}u\left( {I_{0} } \right)} \right)^{2} + \left( {\frac{{\partial \mathop {m_{{I_{2} }} }\limits^{.} }}{{\partial I_{v} }}u\left( {I_{v} } \right)} \right)^{2} } .$$

After substituting various sensitivity coefficients from “Eq. (11)” to “Eq. (16)” in “Eq. (17)” the combined uncertainty in iodine measurement was obtained as “Eq. (18)”:

18 $$u\left( {\mathop {m_{{I_{2} }} }\limits^{.} } \right) = \sqrt {\begin{array}{*{20}l} {\left\{ {\frac{{\mathop {m_{{I_{2} }} }\limits^{.} }}{{M_{c} }}u\left( {M_{c} } \right)} \right\}^{2} + \left\{ {\frac{{\mathop {m_{{I_{2} }} }\limits^{.} }}{T}\left( {1 + \frac{{\mathop {m_{{I_{2} }} }\limits^{.} }}{{M_{c} }}} \right)u\left( {T_{0} } \right)} \right\}^{2} + \left\{ { - \frac{{\mathop {m_{{I_{2} }} }\limits^{.} }}{L}\left( {1 + \frac{{\mathop {m_{{I_{2} }} }\limits^{.} }}{{M_{c} }}} \right)u\left( L \right)} \right\}^{2} + \left\{ { - \frac{{\mathop {m_{{I_{2} }} }\limits^{.} }}{{\left\{ {P_{{tot}} - \left( {\frac{{kT_{0} }}{{\sigma _{v} L}}} \right)\ln \left( {\frac{{I_{0} }}{{I_{v} }}} \right)} \right\}}}u\left( {P_{{tot}} } \right)} \right\}} \\ { + \left\{ {\frac{{\mathop {m_{{I_{2} }} }\limits^{.} }}{{I_{0} \ln \left( {\frac{{I_{0} }}{{I_{v} }}} \right)}}\left( {1 + \frac{{\mathop {m_{{I_{2} }} }\limits^{.} }}{{M_{c} }}} \right)u\left( {I_{0} } \right)} \right\}^{2} + \left\{ { - \frac{{\mathop {m_{{I_{2} }} }\limits^{.} }}{{I_{v} \ln \left( {\frac{{I_{0} }}{{I_{v} }}} \right)}}\left( {1 + \frac{{\mathop {m_{{I_{2} }} }\limits^{.} }}{{M_{c} }}} \right)u\left( {I_{v} } \right)} \right\}^{2} } \\ \end{array} }$$

Using all sensitivities from “Eq. (11)” to “Eq. (16)”, parametric uncertainties from Table 5 and detector output value for iodine absorption after amplification (gain = 50) from Fig. 5,

**Table 5:** Relative uncertainty in lasing specie (iodine) measurement.

	Parameter					
	$I_{0}$	$I_{v}$	$M_{c}$	$T_{0}$	$P_{tot}$	$L$
Measured value, $x_{i}$	I_0_/I_v_ = 6.2 V/1.2 V = 5.2		480 × 10^–3^ mol/s	353 K	2 × 10^4^ Pa	10^–1^ m
Uncertainty, $u\left( {x_{i} } \right)$ (from Table 2)	1.2 × 10^–3^ V	1.2 × 10^–3^ V	1.1 × 10^–2^ mol/s	1.2 K	320 Pa	0.02 × 10^–3^ m
Relative uncertainty = 3%						

$u\left( {\mathop {m_{{I_{2} }} }\limits^{.} } \right)$ is estimated as 3 × 10^–4^ mol s^−1^. Relative uncertainty in iodine measurement is estimated as:

$${\raise0.7ex\hbox{${u\left( {\mathop m\limits^{.}_{{I_{2} }} } \right)}$} \!\mathord{\left/ {\vphantom {{u\left( {\mathop m\limits^{.}_{{I_{2} }} } \right)} {\mathop m\limits^{.}_{{I_{2} }} }}}\right.\kern-\nulldelimiterspace} \!\lower0.7ex\hbox{${\mathop m\limits^{.}_{{I_{2} }} }$}}$$ and is listed in Table 5.

## Results and discussion

 Standard (std.) uncertainty associated with the sensors and detector used in development of DAAS for COIL laser operation and functional optimization measuring fundamental parameters is evaluated in form of *Type-B* uncertainty. Combined std. uncertainty, $u_{C} \left( y \right)$, is the actual measurement uncertainty and is estimated by the root sum square (RSS) of Type A and Type B uncertainties. It is given by $u_{C} \left( y \right) = \sqrt {\left\{ {u_{B} \left( {x_{i} } \right)} \right\}^{2} + \left\{ {u_{A} \left( {x_{i} } \right)} \right\}^{2} }.$

**Figure 6:**
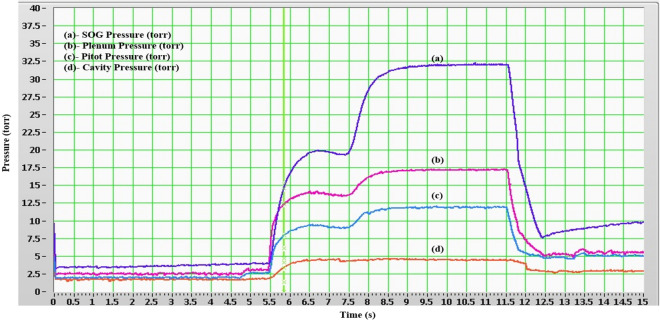
Temporal pressure variations during laser generation.

 Expanded uncertainty U describes the uncertainty limits for enclosing the desired percentage of the population and with 95% confidence interval^15^ it is evaluated by, $U_{95} = ku_{C} \left( y \right)$, multiplication of combined uncertainty and coverage factor *k*_*p*_. For *k*_*95*_*,* coverage value is 2.0. All the std. uncertainty, combined uncertainty, expanded uncertainty and relative combined std. uncertainty, $u_{r} (\% ) = \left\{ {{{u_{C} \left( y \right)} \mathord{\left/ {\vphantom {{u_{C} \left( y \right)} A}} \right. \kern-\nulldelimiterspace} A}} \right\}{{ \times }}100$, have been evaluated for all significant influencing parameters and shown in Table 6. Figure 6 shows temporal pressure variations obtained at different locations of COIL laser system like Singlet Oxygen Generator (SOG), plenum, pitot and cavity during laser generation process (For conversion, 1 torr = 131.6 Pa).

**Table 6:** Expanded and relative uncertainty relevant to sensors/detectors.

**Uncertainty**						
Parameter (sensor)	Theoretical $u_{B} \left( {x_{i} } \right)$ (Type B)	Experimental $u_{A} \left( {x_{i} } \right)$ (Type A)	Combined Std. uncertainty $u_{C} \left( y \right)$	Expanded uncertainty $U_{95} = ku_{C} \left( y \right)$ (for $k_{95}$, coverage value = 2.0)	Parametric values for optimum COIL output $\left( A \right)$	Relative combined Std. uncertainty $u_{r} (\% )$
Pressure (cavity)	32 Pa	0.25 Pa	32 Pa	64 Pa	552.7 Pa (4.2 torr) (from Fig. 6 after conversion from torr to Pa)	5.8%
Temperature	1.2 K	0.03 K	1.2 K	2.4 K	353 K	0.3%
Level	0.09 kPa	–	0.09 kPa	0.18 kPa	4 kPa	2%
EPR	0.003 MPa	–	0.003 MPa	0.006 MPa	1.3** × **10^5^ Pa	2.3%
Flow	0.05 lpm	–	0.05 lpm	0.10 lpm	4.1 lpm	1.2%
Iodine detector	1.2** × **10^–3^ V	–	1.2** × **10^–3^ V	2.4** × **10^–3^ V	24** × **10^–3^ V (1.2 V/50)	5%

Real-time temporal pressure variations at various critical subsystem locations including SOG, plenum, Pitot, cavity and gas/liquid reagent were recorded in the form of a complex pressure plot. It justifies the usefulness of the developed hybrid DAAS system during COIL laser experiments. The sequential operation of various processes has been performed by the DAAS in order to maintain the cavity pressure in the range of 3–6 Torr during laser operation.

The involved complexity is evident from the pressure variation inside the SOG. The first rise in pressure is due to initiating primary nitrogen flow (at 5.3 s) (Ist hump) which effectively compensates chlorine flow (0.45 mol s^−1^) which is started at 7.4 s. At this time the primary flow rate drops from 1.5 to 1.0 mol s^−1^. The secondary nitrogen flow (0.5 mol s^−1^) carrying the lasing medium is initiated at nearly the same time leading to the second pressure rise (IInd hump).

**Figure 7:**
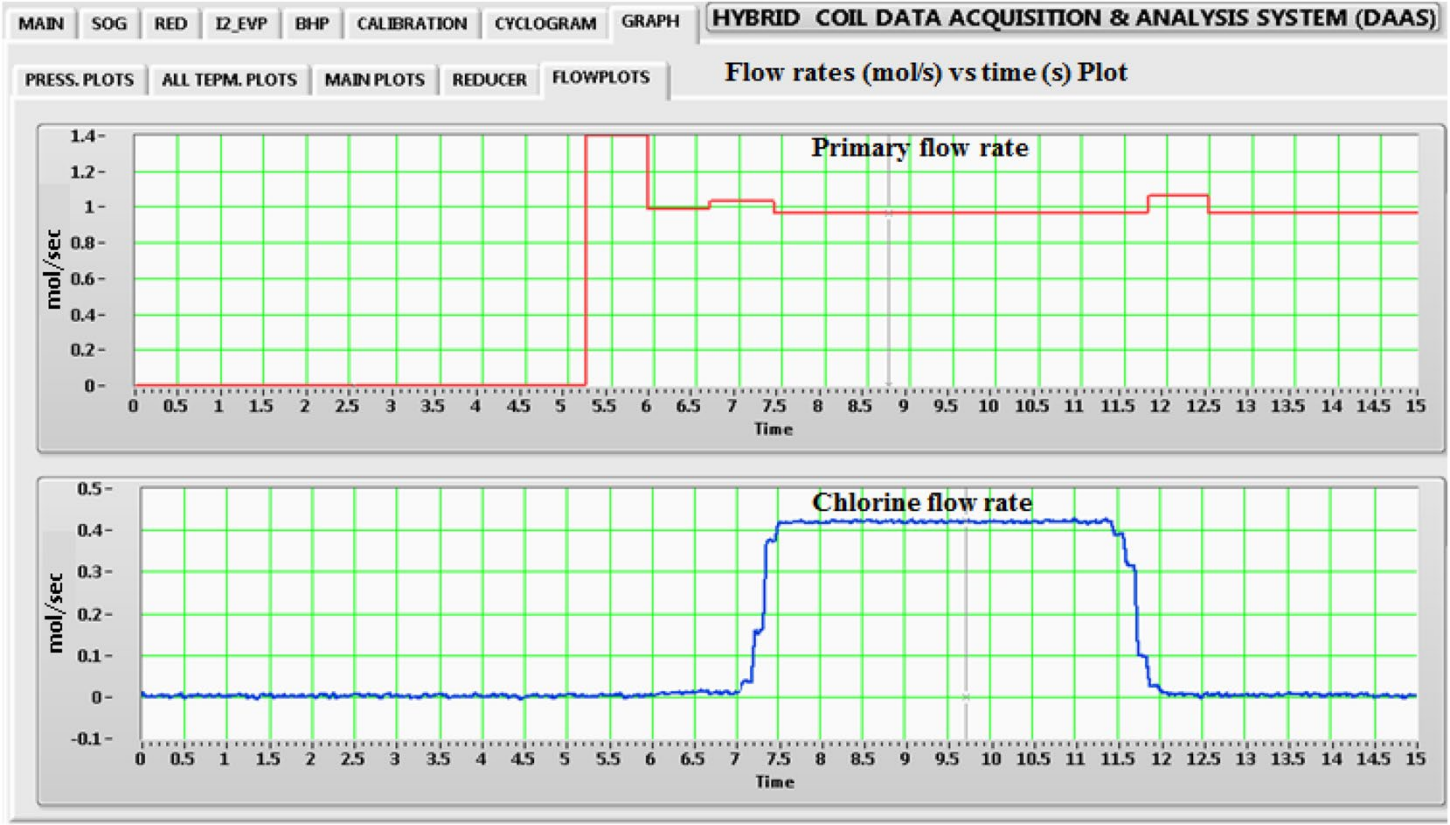
Temporal flow rate variation, flow rate (mol s^−1^) vs. time (s).

The typical stable pressures achieved for SOG, Plenum, Pitot and Cavity are 32 torr, 17 torr, 12 torr and 5.5 torr respectively. In addition, the flow rates for primary Nitrogen and Chlorine also have been obtained directly in *flow plots* (under Graph GUI), Fig. 7. It clearly verifies the start of flow of Primary Nitrogen and Chlorine at 5.3 s and 7.4 s respectively.

Relative uncertainty of all the derived parameters, degree of freedom,

$$v_{i} \approx \frac{1}{2}\left[ {\frac{{\Delta u\left( {x_{i} } \right)}}{{u\left( {x_{i} } \right)}}} \right]^{ - 2} \approx \frac{1}{2}\left[ {u\left( r \right)} \right]^{ - 2} ,$$

and their coverage factor (using t-distribution table) are listed in Table 7.

**Table 7:** Relative uncertainty in derived parameter measurement.

Derived Parameter	Relative uncertainty $u\left( r \right)$	Degree of Freedom (for Type B evaluation) $v_{i}$	Coverage Factor (From t distribution table & 95% CL)
**Flow rate**			
Primary nitrogen	0.7%	10,204	1.961
Secondary nitrogen	2.3%	945	1.963
Chlorine	0.4%	31,250	1.960
Lasing specie concentration	3%	556	1.964

### Overall uncertainty evaluation for the DAAS system

From the analyses of the various sources of uncertainty in DAAS used for COIL, employing both uncertainty and sensitivity of sensors, it is clear that it is viable to correctly examine the DAAS system performance and its efficacy. Since no direct relation exists between the sensor architecture, display and control device in development of DAAS, hence instead of Monte-Carlo method^34^ the GUM tool has been utilized^11,35,36^ for uncertainty analysis.

Detailed uncertainty analysis using both *Type-A* and *Type-B* methods have been carried out for fundamental as well as derived parameters relevant to COIL laser and summarized in Tables 4 and 6 respectively. It is clear that the combined uncertainty in both pressure and temperature acquisition using developed DAAS is nearly identical with *Type-B* uncertainty (i.e. influence of *Type-A* uncertainty is negligible). Hence, in order to avoid the effect of double count problem of uncertainty components, effect of *Type-B* uncertainty is considered as a prime component for evaluating combined uncertainty.

Relative combined std. uncertainty, *U(total)* for developed DAAS is evaluated^19^ as indicated as “Eq. (19)”:

19 $$U\left( {total} \right) = \sqrt {\left\{ {u\left( {r,\Pr ess} \right)} \right\}^{2} + \left\{ {u\left( {r,Temp} \right)} \right\}^{2} + \left\{ {u\left( {r,Level} \right)} \right\}^{2} + \left\{ {u\left( {r,EPR} \right)} \right\}^{2} + \left\{ {u\left( {r,Flow} \right)} \right\}^{2} + \left\{ {u\left( {r,Iodine} \right)} \right\}^{2} } = 8.3\% .$$

The quantification of *U*(*total*) for the system is critical in terms of assessing relative correctness (closeness) of the data indicated by the DAAS to the true values of critical parameters of the COIL system. In order to be able to accurately optimize a multidisciplinary system such as COIL involving high speed flow, it is imperative that overall uncertainty of the system is within 5–10% ranges. It is all the more essential as in COIL, subsystems are capable of producing significant mutual influences creating spiraling effect and rendering the system non functional since the operating band for such flow medium lasers is apparently quite narrow. Thus, a detailed assessment of *U(total)* is useful for optimal operation of complex gas laser system such as COIL. It is evident from the above calculations, Tables 6 and 7 that measurement uncertainty of developed DAAS is mainly governed by instabilities of pressure sensor. Hence performance of DAAS system is much more sensitive to changes in pressure sensor than changes in any other fundamental or derived parameter uncertainty. Hence, it underlines the need for careful selection of pressure sensor and its precise calibration to reduce *U(total)*. The use of remote DAAS facilitates parametric and system uncertainty assessment by circumventing manual measurement/ observation and enabling carrying out repeated experimentation for generating exhaustive data.

## Conclusion

A dedicated 150 channels (64AI, 64DO, 5AO, 17DI) DAAS has been designed and developed using LabVIEW application software to fulfill the operation, acquisition and analysis requirement of flowing medium high power (2.3 mol/s) COIL laser. Precise operational control of DAAS has been achieved using accurate and reliable data from the sensor system. This DAAS is used for various experiments of COIL laser and related technologies which proves its efficacy.

GUM algorithm approaches have been utilized for evaluation of uncertainty of the developed DAAS system. This would potentially provide significant insights for improving the developed system by determining detailed uncertainty for both fundamental as well as derived parameters. Based on the uncertainty analysis it is evident that *Type-B* uncertainty computation of standard uncertainty is more reliable than *Type-A* uncertainty. It is inferred that DAAS system performance is primarily governed by pressure sensor uncertainty. The pressures at different locations of COIL system are further controlled by the flow rates of different gas constituents like N_2_, Cl_2_ and I_2_. The laser power achieved is essentially the measure of overall system performance and the estimated total variation is 8.3%. More than 200 experiments were carried out successfully and controlled safe operation of high power COIL laser system was achieved by using the developed DAAS system after obtaining accurate and reliable data from the sensor/detector system. This enables prediction of overall system uncertainty potentially extendable to other similar laser systems involving subsystems with mutual interdependencies together being distributed over a significantly large laboratory space. To the best of our understanding, this is amongst the first attempt to report the uncertainty evaluation of a highly complex Chemical Oxygen Iodine Laser system.

Enhanced decision-making in optimizing a complex supersonic flowing medium gas laser has been achieved by developing a suitable hybrid DAAS. The existing approaches to uncertainty have been innovatively utilized to not only predict the overall system uncertainty but also the most crucial influencing parameter i.e. Pressure. This in a way presents a measure of the reliability of system performance in a battlefield scenario, enabling the prediction of the potential effect on the target reasonable precision.

## Data Availability

All data generated or analysed during this study are included in this published article [and its supplementary information files].

## References

[1] Carroll DL (2011). Overview of high energy lasers: Past, present and future?. Proc. AIAA. Plasmadyn. Laser Conf..

[2] Witteman, W. J. *The CO*_*2*_* Laser*. (Springer, 1987)*.*10.1007/978-3-540-47744-0.

[3] Behrens, W. H. & Lohn, P. D., Hydrogen and deuterium fluoride chemical lasers. In *Gas Lasers *341–367 (CRC Press, 2007). 10.1201/b13628.

[4] McDermott WE, Pchelkin NR, Benard DJ, Bonsek RR (1978). An electronic transition chemical laser. Appl. Phys. Lett..

[5] Yoshida S, Endo M, Sawano T, Amano S, Fuji H (1999). High-pressure, high-efficiency operation of a chemical oxygen-iodine laser. Appl. Phys. Lett..

[6] Carroll DL (2000). High performance chemical iodine laser using nitrogen diluents for commercial applications. IEEE J. Quantum Electron..

[7] Tyagi RK, Rajesh R, Singhal G, Mainuddin DAL, Endo M (2003). Supersonic COIL with angular jet singlet oxygen generator. J. Opt. Laser Technol..

[8] Endo M, Osaka T, Takeda S (2004). High-efficiency chemical oxygen-iodine laser using a streamwise vortex generator. Appl. Phys. Lett..

[9] Rybalkin V, Katz A, Barmashenko BD, Rosenwaks S (2004). Nearly attaining the theoretical efficiency of supersonic chemical oxygen-iodine lasers. Appl. Phys. Lett..

[10] *The Expression of Uncertainty and Confidence in Measurement.* M3003, Edition 3, UKAS (2012). ISBN 978-0-948926-30-3.

[11] *Guide to the Expression of Uncertainty in Measurement.* International Organization for Standardization, Geneva. & (2008). JCGM 100:2008 (1995).

[12] *BIPM—International Bureau of Weights and Measures Joint Committee for guides in metrology (JCGM) guide: Evaluation of measurement, Official version* http://www.bipm.org (2015).

[13] Ellison, S. L. R. & Williams, A. Quantifying uncertainty in analytical measurement. *Eurachem/CITAC guide*, 3rd ed. http://www.eurachem.org (2012). ISBN 978-0-948926-30-3.

[14] Moffat, R. J. Describing the uncertainties in measurement results. In *Experimental Thermal and Fluid Science*, vol. 1, 3–17 (Elsevier Science Publishing Co., 1988). https://citeseerx.ist.psu.edu/viewdoc/download?doi=10.1.1.466.2017&rep=rep1&type=pdf.

[15] Mainuddin SG, Tyagi RK, Maini AK (2012). Diagnostics and data acquisition for chemical oxygen iodine laser. IEEE Trans. Instrum. Meas..

[16] Otomanski P, Szlachta A (2008). The evaluation of expanded uncertainty of measurement results in direct measurement using the LabVIEW environment. Meas. Sci. Rev..

[17] Annamalai K, Visvanathan K, Sriramulu V, Bhaskaran KA (1998). Evaluation of the performance of supersonic exhaust diffuser using scaled down models. Exp. Therm. Fluid Sci..

[18] Furman D, Barmashenko BD, Rosenwaks S (1999). Diode-laser based absorption spectroscopy diagnostics of a jet-type generator for chemical xygen iodine lasers. IEEE J. Quantum Electron..

[19] Yu L, Teng G, Riskowski GL, Xu X, Guo W (2018). Uncertainty analysis of a web-based data acquisition system for poultry management with sensor networks. Engenharia Agrícola Jaboticabal..

[20] Willink R (2013). An improved procedure for combining type A and type B components of measurement uncertainty. Int. J. Metrol. Qual. Eng. EDP. Sci..

[21] Mainuddin TRK, Rajesh R, Singhal G, Dawar AL (2003). Real-time data acquisition and control system for a chemical oxygen-iodine laser. J. Meas. Sci. Technol. IOP. UK.

[22] Collins, D. Wireless data acquisition in Flight Test Networks. In *3rd European Telemetry and Test Conference* 225–232 (Curtiss-Wright Corporation, 2016).

[23] Cranley, N. Experimental Investigation of Wireless technologies for data acquisition, ITC.

[24] Salvadori F, Gehrke CS, de Campos M, Sausen PS, Olivieria AC (2012). A hybrid network architecture applied to smart grid. Int. J. Comput. Netw. Technol..

[25] Sausen, P. *et al*. Design, development and implementation of a hybrid network with smart sensors and power line communication for monitoring of underground electricity substation. In *Second International Conference on Advance Communication and Computation*. 130–135 (2012).

[26] Diego F, Garcia J, Esteban B, Merello P (2015). Design of a hybrid (wired/wireless) acquisition data system for monitoring of cultural heritage physical parameters in the smart cities. Sensors..

[27] Mirabella R, Brischetto M (2011). A hybrid wired/wireless networking infrastructure for greenhouse management. IEEE Trans. Instrum. Meas..

[28] Sheng X, Minrui F, Haikuan W (2016). Design of hybrid wired/wireless fieldbus network for turbine power generation system. Inf. MDPI..

[29] Mainuddin, Singhal, G. & Dawar, A. L. *Sensors and Measurement Techniques for Chemical Gas Lasers*. *International Frequency Sensor Application (IFSA, 2014) Publishing.* Barcelona, Spain. ISBN-13: 978-84-617-1865-8.

[30] Grzegorz K (2019). Multichannel data acquisition system for GEM detectors. J. Fusion Energy.

[31] Dohare, R. K., Mainuddin & Singhal, G. Hybrid data acquisition system for flowing medium lasers. *Defence Sci. J.* **70**(3), 285–291. 10.14429/dsj.70.14902. (2020).

[32] Dohare, R. K., Mainuddin, K. S. & Singhal. G. Data Acquisition system for chemical iodine generation suitable for flowing medium chemical oxygen iodine laser. *Defence Sci. J.* **71**(6), 798–806 (2021). 10.14429/dsj.71.17026.

[33] Dohare, R. K., Mainuddin & Singhal. G. Real time flow control system for precise gas feed in COIL. *Defence Sci. J.* **72**(1), 91–97 (2022). 10.14429/dsj.72.17079.

[34] Beherens R (2010). Uncertainty in external dosimetry: Analytical vs. Monte Carlo method guide to the expression of uncertainty in measurement. Radiat. Prot. Dosim..

[35] Farrance & Frenkel, R. Uncertainty of measurement: A review of the rules for calculating uncertainty components through functional relationships. *Clin. Biochem. Rev.* **33**(2), 49–75 (2012). PMC3387884 22896744

[36] Joint Committee for Guides in Metrology, Evaluation of measurement data—Supplement 1 to the Guide to the expression of uncertainty in measurement, Propagation of distributions using a Monte Carlo method (2006).

